# 

**Published:** 1791

**Authors:** 


					MEDICAL FACTS
AND
OBSERVATIONS.
' /
v'OL. X. V--2Z*
t ?
f /. L-f. I II M
O-r' w-'
/
) - f ft* i i . i
f i C.. &-{.-?
f \
/I f / / ^ ?
if - l ?. c -? ?'( r c

				

